# Unravelling heterogeneous effects of cancer‑associated fibroblasts on poor prognosis markers in breast cancer EM‑G3 cell line: *In vitro*‑targeted treatment (anti‑IL-6, anti‑VEGF-A, anti‑MFGE8) based on transcriptomic profiling

**DOI:** 10.3892/or.2023.8662

**Published:** 2023-11-15

**Authors:** Lukáš Urban, Štepán Novák, Matúš Čoma, Barbora Dvořánková, Lukáš Lacina, Jana Šáchová, Miluše Hradilová, Petra Svatoňová, Michal Kolář, Hynek Strnad, Jana Březinová, Karel Smetana, Peter Gál, Pavol Szabo

**Affiliations:** 1 Department of Pharmacology, Faculty of Medicine, Pavol Jozef Šafárik University in Košice, 040 11 Košice, Slovak Republic; 2 Department for Biomedical Research, East-Slovak Institute of Cardiovascular Diseases, Inc., 040 11 Košice, Slovak Republic; 3 Institute of Anatomy, First Faculty of Medicine, Charles University, 128 00 Prague, Czech Republic; 4 Department of Otorhinolaryngology, Head and Neck Surgery, First Faculty of Medicine, Charles University and University Hospital Motol, 150 06 Prague, Czech Republic; 5 BIOCEV, Charles University, First Faculty of Medicine and Faculty of Sciences, 252 50 Vestec, Czech Republic; 6 Department of Dermatovenereology, General University Hospital in Prague and First Faculty of Medicine, Charles University, 128 00 Prague, Czech Republic; 7 Laboratory of Genomics and Bioinformatics, Institute of Molecular Genetics, Czech Academy of Sciences, 142 20 Prague, Czech Republic; 8 Cytogenetic Laboratory, Institute of Hematology and Blood Transfusion, 128 00 Prague, Czech Republic; 9 Department of Pharmacognosy, Faculty of Pharmacy, Comenius University in Bratislava, 832 32 Bratislava, Slovak Republic; 10 Prague Burn Center, Third Faculty of Medicine, Charles University, 100 34 Prague, Czech Republic; 11 Insitute of Neurobiology, Biomedical Research Center of the Slovak Academy of Sciences, 040 01 Košice, Slovak Republic

**Keywords:** epithelial-mesenchymal interaction, cell differentiation, tumor microenvironment, breast cancer, neutralizing antibody

## Abstract

Breast cancer is the most frequently diagnosed cancer in women worldwide. Although dramatically increased survival rates of early diagnosed cases have been observed, late diagnosed patients and metastatic cancer may still be considered fatal. The present study's main focus was on cancer-associated fibroblasts (CAFs) which is an active component of the tumor microenvironment (TME) regulating the breast cancer ecosystem. Transcriptomic profiling and analysis of CAFs isolated from breast cancer skin metastasis, cutaneous basal cell carcinoma, and squamous cell carcinoma unravelled major gene candidates such as *IL6, VEGFA* and *MFGE8* that induced co-expression of keratins-8/-14 in the EM-G3 cell line derived from infiltrating ductal breast carcinoma. Western blot analysis of selected keratins (keratin-8, −14, −18, −19) and epithelial-mesenchymal transition-associated markers (SLUG, SNAIL, ZEB1, E-/N-cadherin, vimentin) revealed specific responses pointing to certain heterogeneity of the studied CAF populations. Experimental *in vitro* treatment using neutralizing antibodies against IL-6, VEGF-A and MFGE8 attenuated the modulatory effect of CAFs on EM-G3 cells. The present study provided novel data in characterizing and understanding the interactions between CAFs and EM-G3 cells *in vitro*. CAFs of different origins support the pro-inflammatory microenvironment and influence the biology of breast cancer cells. This observation potentially holds significant interest for the development of novel, clinically relevant approaches targeting the TME in breast cancer. Furthermore, its implications extend beyond breast cancer and have the potential to impact a wide range of other cancer types.

## Introduction

Breast cancer is the most frequently diagnosed cancer in women worldwide. In the European Union, the incidence ratio encounters over 400,000 new cases every year, with a 5-year survival rate approaching ~96% in early diagnosed disease (1). A comprehensive approach involving surgery, radiation therapy, (neo)adjuvant chemotherapy, hormone therapy, targeted therapy, and immunotherapy (2,3) has led to a dramatic increase in the 5-year survival rates compared with those recorded in the 1980s (73% in Europe and 84% in the USA) (4). Notably, substantial progress has been made in the field of immunotherapy for the treatment of poor-prognosis triple-negative breast cancer (5–7). However, further studies need to be conducted to improve the patient care, in particular in cases with late diagnosed and metastatic cancer that may still be considered fatal (8). Despite newly characterized gene-profiling models predicting disease outcome (9), the treatment protocols still heavily rely on traditional histopathologic examination (type, grade) along with the detection of the expression of the three key receptors (estrogen receptor α, progesterone receptor and human epidermal growth factor receptor 2) (10).

As rapidly the knowledge of biological processes underlying the growth and spread of cancer has expanded, the tumor microenvironment (TME) has been also identified as a crucial part comprising non-cellular [extracellular matrix (ECM), soluble and physical factors produced mainly by non-cancer cells] and cellular components (immune and non-immune cells of the local and distant origin) (11) with specific nuances for breast cancer (12). The intricate interplay between cancer cells and their microenvironment plays a crucial role in shaping the fundamental characteristics of tumors including cancer cell proliferation, propagation and response to therapy (13). Fibroblasts may be considered one of the most abundant and critical cell types in the tumor stroma. In addition to the ECM, they also produce several signaling/regulatory molecules (14). Fibroblasts also produce pro-inflammatory IL-6 promoting the growth and radio resistance of breast cancer cells (15). Conversely, inflammatory cytokines that are also produced by cancer cells, host immune and stromal cells induce the activation of fibroblasts (16). It is important to acknowledge that although activated fibroblasts, simply stated by the authors, as cancer-associated fibroblasts (CAFs), they constitute a heterogeneous group of tumor stromal cells derived from different precursors (for example, adipocytes, bone-marrow-derived fibrocytes, endothelial cells, mesenchymal stem cells, pericytes, stellate cells, or smooth muscle cells) (17). It was demonstrated that through production of soluble factors, such as cytokines and chemokines (18,19) and/or a bioactive ECM scaffold (20) CAFs strongly influence surrounding cells, resulting in tumor progression/metastasis, enhanced drug resistance and angiogenesis (21). In line with this evidence, the previous comparative analysis of CAFs was isolated from different tumors (breast cancer skin metastasis, cutaneous basal cell carcinoma, squamous cell carcinoma arising from oral cavity mucous membrane, and melanoma) and found that the effect of CAFs on EM-G3 cells is rather tumor unspecific (each studied culture of CAFs induced co-expression of keratins-8/-14 in the EM-G3 cells) (22). Therefore, it was further hypothesized that the transcriptomic comparison of the CAF gene expression profile could select overlapping candidate genes that may have the potential to target the breast cancer microenvironment.

EM-G3 cells were derived from a primary lesion of human infiltrating ductal breast carcinoma (23). The rationale behind the use of a premalignant population of common progenitors of luminal and myoepithelial cells (immortalized in an early stage of tumorigenesis) the model of the present study was to evaluate whether/how fibroblasts shift the fate of EM-G3 cells. In particular, the authors were interested in whether the originally less invasive behaviour would change by co-culturing with CAFs and whether these changes may be attenuated by a combination of neutralizing antibodies. For the initial step, an extensive transcriptomic profiling was initiated and analysis of CAFs isolated from studied tumors. The cluster analysis based on transcriptomic datasets has the potential to unravel disease phenotypes missed by histopathological examination, and such an approach is frequently used in cancer research and led to the discoveries of novel tumor subtypes (24), including breast cancer (25,26). Indeed, major gene candidates were identified including *IL6, VEGFA* and *MFGE8* that may play important role in the regulation of TME in breast cancer. In line with this evidence, the present *in vitro* study revealed the importance of the studied proteins in modulating the epithelial-mesenchymal interactions between EM-G3 breast cancer cells and CAFs prepared from different tumors.

## Materials and methods

### Tissue samples and isolation of primary cultures of fibroblasts

Samples of normal skin (human dermal fibroblasts; HDF) were obtained from the Department of Aesthetic Surgery, Third Faculty of Medicine, Charles University and University Hospital Kralovske Vinohrady, (Prague, Czech Republic). Samples of basal cell carcinoma (T1N0M0) [basal cell carcinoma fibroblast (BCCF)] and of squamous cell carcinoma of oral cavity (T3N2M0) ([squamous cell carcinoma fibroblast (SCCF)] were obtained from the Department of Otorhinolaryngology, Head and Neck Surgery, First Faculty of Medicine (Prague, Czech Republic). Fibroblasts from a cutaneous metastasis of breast cancer (ductal infiltrating carcinoma, T4bN0M1) [breast cancer metastasis fibroblast (BCMF)] were isolated at the Department of Dermatovenereology, First Faculty of Medicine, Charles University (Prague, Czech Republic). Fibroblasts were isolated, cultured and expanded as previously described (27–29). Phenotype characterization of the obtained fibroblasts was evaluated by detection of vimentin (+), fibronectin (+), FAP (+), PDGFR-α/β (+), high molecular weight keratins (−), tyrosinase (−), CD45 (−), and MiTF (−) (not shown). Cells were isolated from tissue samples obtained with informed consents of the patients respecting Declaration of Helsinki approved by Ethical Committee of General University Hospital (approval nos. 8/11, 15/15 and 7/21; Prague, Czech Republic) and Ethical Committee of University Hospital Královské Vinohrady (approval no. 100/1947/2005; Prague, Czech Republic). Written informed consent was provided by all patients.

### Human breast cancer EM-G3 cell line

The EM-G3 cell line (23) was isolated from infiltrating ductal breast carcinoma (grade II, pT1cN0M0, ER^+^, PR^+^, and HER2^+^), which was profoundly characterized using the proteomic approach (30,31). Stability of the cell line genome was verified by multicolour fluorescence *in situ* hybridization (mFISH). The analysis confirmed a stable diploid genome with several genetic changes (Fig. S1). Karyotype was re-evaluated in 15 metaphasesas:44,XX,der(3)t(3;9)(q26;?),der(6)t(6;15)(p21;q12),der(12)t(12;12)(p13.2;q?)dup(12)(q21.2q21.3)dup(12)(q21.2q21.3),der(13)t(13;20)(p11;?),−15,−20. The second clone was observed in five metaphases with identical chromosomal aberrations with additional derived chromosome 7 identified as: 45,idem,+der(7)t(7;12)(q11;?).

### Cultivation of fibroblasts and EM-G3 cells

All fibroblasts HDF 2nd passage, BCCF 6th passage, SCCF 5th passage and BCMF 4th passage were stored in liquid nitrogen and cultivated in Dulbecco's modified Eagle's medium (DMEM; Biochrom, Ltd.) supplemented with 10% fetal bovine serum (FBS; Biochrom, Ltd.) and cultured at 37°C and 5% CO_2_. EM-G3 28th passage was stored in liquid nitrogen, cells were cultured in keratinocyte medium (keratinocyte culture medium: DMEM and F12 medium 1:1 (Biochrom, Ltd.) with 10% FBS enriched with insulin, choleratoxin, hydrocortisone, and epidermal growth factor (32).

### Evaluation of the senescent phenotype of the studied fibroblasts

The senescence-associated phenotype of CAFs appears to stimulate tumor growth and metastasizing (33). Therefore, the senescence of the tested fibroblasts was evaluated by detection of senescence-associated enzyme β-galactosidase (SAβGal) using a Senescence Cells Histochemical Staining kit (cat. no. CS0030; Sigma-Aldrich; Merck KGaA) according to the manufacturer's instructions. β-Galactosidase activity is detectable in senescent cells, but not in quiescent, immortal, or tumor cells.

### RNA isolation and genome-wide transcriptome analysis using RNA sequencing

Total RNA was isolated from CAFs and HDF using RNeasy Micro Kit (Qiagen Sciences, Inc.) according to the procedure for animal cells. The quantity of RNA was measured spectrophotometrically in NanoDrop ND-1000 (Thermo Fisher Scientific, Inc.) and Agilent 2100 Bioanalyser (Agilent Technologies, Inc.). All further analyzed RNA samples had RIN >8. Illumina HumanHT-v3 Expression BeadChip (Illumina, Inc.) was used for microarray analysis following the standard protocol – 150 ng of total RNA was amplified with Illumina^®^TotalPrep RNA Amplification kit (cat. No. AMIL1791; Ambion; Thermo Fisher Scientific, Inc.) and 750 ng of amplified RNA was hybridized on the chip according to the manufacturer's procedure and scanned using BeadArray Reader (Illumina, Inc.).

The resulting data were processed using the limma package (version 3.22.0) (34) within the R/Bioconductor environment with probe annotation provided by the HumanHT-12_V3_0_R3_11283641_A.bgx manifest file (Illumina, Inc.). The data were background corrected using the normal-exponential mixture method, quantile normalized, and log_2_-transformed to stabilize variance. The fitted statistical model assumed sample groups according to the cell type (HDF, BCCF, SCCF and BCMF). F-test with residual mean squares moderated between genes was performed to detect differentially expressed genes. Only genes with concordant changes in comparisons between three CAF types and HDF (|log_2_FC| >0.8) and FDR <0.005 were considered differentially expressed (Tables SI and SII).

Functional enrichment analysis was performed using the Enrichr web server (35) with respect to the Elsevier Pathway Collection of manually annotated pathways. Only pathways with at least three assigned upregulated genes were considered, top five pathways are displayed in Table SIII.

The microarray data are available in MIAME (Minimum Information About a Microarray Experiment) compliant format in the ArrayExpress database under accession E-MTAB-12994 (https://www.ebi.ac.uk/biostudies/arrayexpress/studies/E-MTAB-12994) (36).

### ELISA

Concentrations of IL-6 (cat. no. QK206), VEGF-A (cat. no. DVE00) and MFGE8 (cat. no. DFGE80) were measured in conditioned media (CM) (after 24 h culturing) derived from the studied CAFs by enzyme-linked immunosorbent assay (ELISA, R&D Systems, Inc.) according to the procedure provided by the manufacturer. All experiments were performed in two biological replicates. ELISA kits were used to measure the concentrations of gene products (IL-6, VEGF-A and MFGE8) in the CM of CAFs (Table I).

### Co-cultures of fibroblasts and EM-G3 cells

i) Direct co-cultivation: HDFs, BCCFs, SCCFs and BCMFs were seeded on coverslip at 1,000 cells/cm^2^ density together with EM-G3 cells at 5,000 cells/cm^2^ density and cultured in keratinocyte medium for 6 days. Culture medium was changed every second day. The coverslips were then washed three times with PBS, dried, and stored frozen at −20°C for immunocytochemistry. ii) Culture of EM-G3 cells in CM derived from fibroblasts: A total of 15 ml of keratinocyte medium was applied to 75-cm^2^ flasks with confluent growth of fibroblasts (HDFs, BCCFs, SCCFs and BCMFs) and cultured for 24 h at 37°C and 5% CO_2_. Subsequently, CM was collected, centrifuged (4°C, 500 × g, 5 min) and filtered to remove any floating cells. The CM was then either stored at −20°C for ELISA analysis or used fresh for co-culture experiments with EM-G3 cells. EM-G3 cells were seeded on the bottom of six-well plates with [used for immunofluorescence (IF)] or without [used for western blot (WB) analysis] coverslips at the density of 5,000 cells/cm^2^. The next day, the culture medium was changed to CM and replaced every other day. The coverslips with EM-G3 cells were then washed three times with PBS, dried, and stored frozen at −20°C for IF staining. For WB analysis, cells were washed with cold PBS, scratched, and collected into falcon tubes. iii) Culture of EM-G3 cells in media supplemented with human recombinant proteins: Three commercially available human recombinant proteins, IL-6, VEGF-A, and MFGE8 (R&D Systems, Inc.), were (either individually or in combination) added to keratinocyte medium (Table II). The concentration of recombinant proteins was selected and set at 100-fold based on the ELISA test results (Table I) of the CM produced by fibroblasts (0.1 ng/ml for IL-6, 0.5 ng/ml for VEGF-A, and 1 ng/ml for MFGE8). iv) Culture of EM-G3 cells supplemented with blocking antibodies: The activities of IL-6, VEGF-A and MFGE8 (either individually or in combination) in co-cultures were blocked using commercially available antibodies (IL-6, VEGF-A; R&D Systems, Inc. and MFGE8; Exbio Antibodies) used at the following concentrations (1,000-times more than measured by ELISA in CM derived from CAFs): 10 ng/ml of anti-IL-6; 50 ng/ml of anti-VEGF-A; 100 ng/ml of anti-MFGE8; (Table II). The specificity of the reaction and exclusion of an effect of interaction of Fc fragment of the antibody with Fc receptors on epithelial cells was tested by using unspecific antibodies as controls (anti-thyroglobulin, rabbit polyclonal, and mouse monoclonal; all Dako; Agilent Technologies, Inc.).

### IF analysis of cultured cells

Cells seeded on glass coverslips were fixed with 2% buffered paraformaldehyde (pH 7.2) at room temperature for 5 min and washed with PBS. Cells were permeabilized by exposure to Triton X-100 (Sigma-Aldrich; Merck KGaA), and sites for the antigen-independent binding of antibodies were blocked by porcine serum albumin at room temperature for 30 min (Dako; Agilent Technologies, Inc.). Commercial antibodies that were used were diluted as recommended by the suppliers mentioned in Table III. Cell nuclei were counterstained with DAPI (0.1 mg/ml) at room temperature for 1 min (Sigma-Aldrich; Merck KGaA). All specimens were mounted to Vectashield (Vector Laboratories, Inc.) and inspected by an Eclipse 90i microscope (Nikon Corp.) equipped with filterblocks for FITC, TRITC and DAPI, and a black and white (colors of each channel, RGB, are artificially added during postprocessing) Cool-1300Q CCD camera (Vosskühler). Data were analyzed using the LUCIA 5.1 computer-assisted image analysis system (Laboratory Imaging s.r.o.).

### WB analysis of cultured cells

Protein lysate preparation took place after centrifugation (4°C, 500 × g, 5 min), cell pellets were dissolved in Laemmli sample buffer (100 mM TRIS-HCl, 10% glycerol, 2% SDS, pH~6.8) enriched with inhibitors of proteases and phosphatases (both from Sigma-Aldrich; Merck KGaA). Following sonication (QSonica, 40% amplitude, 15 sec), samples were kept on ice for 20 min and centrifuged (4°C, 10,000 × g, 10 min). Protein lysates were pipetted into fresh Eppendorf tubes and stored at −20°C. To assess protein concentration in the prepared lysates, the BCA protein assay was carried out (Pierce™ BCA Protein Assay Kit, Thermo Fisher Scientific, Inc.). WB analysis: samples (10 µg of protein loaded per lane) were quickly boiled (95°C, 5 min) and separated in SDS-PAGE 10% Bis-Tris gel. Afterwards, proteins were electroblotted to PVDF membrane using the iBlot 2 dry blotting system (Thermo Fisher Scientific, Inc.). Following quick washing in TBS-T (0.1% Tween in Tris-buffered saline), membranes were blocked for 1 h at room temperature in 5% NFDM/BSA (non-fat dry milk/bovine serum albumin) TBS-T buffer. After overnight incubation with primary antibodies at 4°C, membranes were washed three times with TBS-T and incubated with appropriate HRP-conjugated secondary antibody for 1 h at room temperature. Finally, chemiluminescent signals from immunoblots were acquired in the iBright FL1500 Imaging System (Thermo Fisher Scientific, Inc.) using ECL (SuperSignal West Pico PLUS chemiluminescent Substrate; Thermo Fisher Scientific, Inc.). β-Actin was used as loading control. The complete list of antibodies used for WB analysis can be found in Table IV.

## Results

### Histochemical and IF analysis of fibroblasts

**Figure 1.**
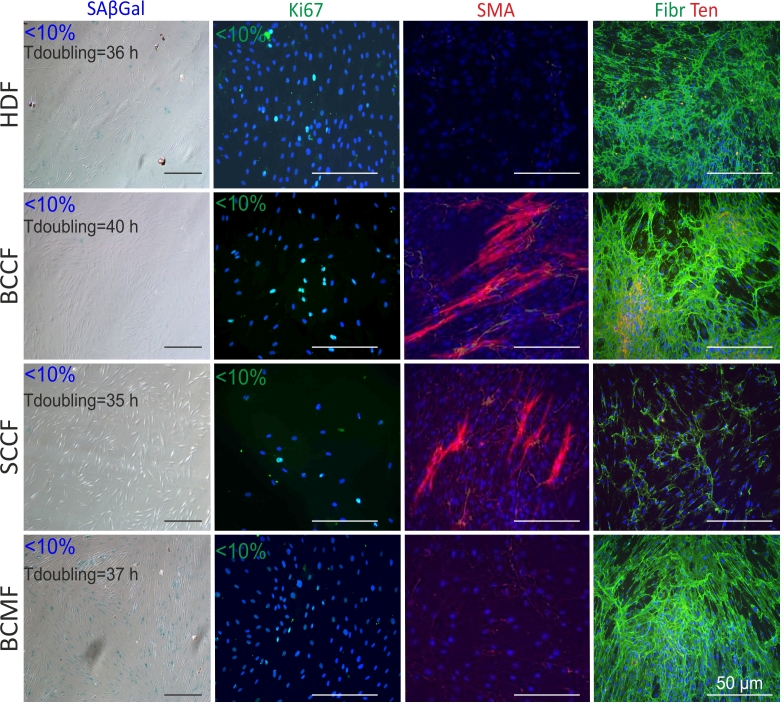
Immunohistochemical analysis of HDFs, BCCFs, SCCFs and BCMFs. Presence of fibronectin (Fibr) and α-SMA. Cell nuclei were counterstained with DAPI. Scale bar, 100 µm; magnification, ×200. HDFs, human dermal fibroblasts; BCCFs, basal cell carcinoma fibroblasts; SCCFs, squamous cell carcinoma fibroblasts; BCMFs, breast cancer metastasis fibroblasts; SMA, smooth muscle actin.

No differences in duplication time between HDFs and CAFs were detected (Fig. 1). Although BCMFs exhibited the strongest histochemical reaction to SAβGal among the examined cells, the ratio of positive cells remained <10% in all studied fibroblasts. Similarly, expression of proliferation marker Ki67 remained weak. The presence of myofibroblasts with high expression of α-smooth muscle actin was predominantly detected in BCCF cultures, while SCCF cultures exhibited lower quantities of myofibroblasts. By contrast, the weak/no expression of α-smooth muscle actin fibers in HDF and BCMF cultures indicated absence of myofibroblasts. High production of fibronectin was observed in all types of cultured fibroblasts except for SCCFs. Expression of tenascin-C, another ECM molecule, was observed only in the culture of BCCFs.

### Transcriptomic analysis of fibroblasts

**Figure 2.**
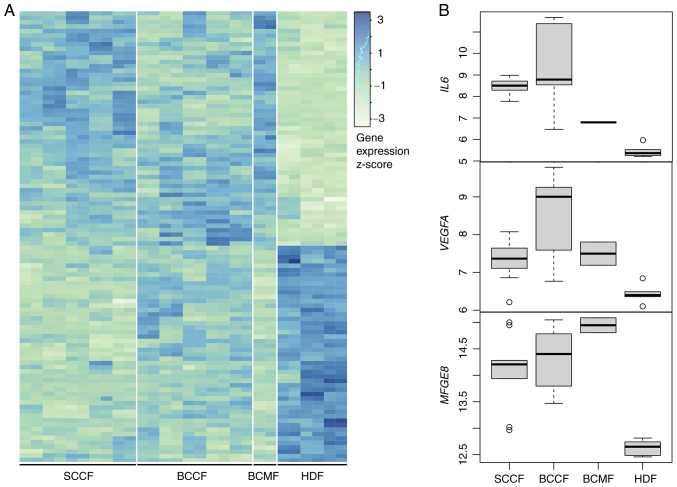
Transcriptomic analysis of HDFs, BCCFs, SCCFs and BCMFs. (A) The heatmap displays expression profiles of all differentially expressed genes with concordant changes between CAFs and HDFs. (B) The expression profiles of the selected candidate genes *IL6, VEGFA*, and *MFGE8* are shown in boxplots. HDFs, human dermal fibroblasts; BCCFs, basal cell carcinoma fibroblasts; SCCFs, squamous cell carcinoma fibroblasts; BCMFs, breast cancer metastasis fibroblasts.

A transcriptomic analysis was performed to identify genes that are dysregulated in all CAF groups (BCCF, SCCF and BCMF) with respect to the normal HDF fibroblasts (Fig. 2A). A total of 53 significantly upregulated genes were detected (Table SI) with significant enrichment of signaling pathways listed in Table SIII. Pathways related to cancer signalling, immune cell infiltration of tumor, and blood vessel sprouting were among the pathways most enriched with the upregulated genes. Among them, the *IL6, VEGFA* and *MFGE8* genes were the most prominent (as evidenced by increased protein expressions evaluated by ELISA); thus, the authors focused on investigating these genes in detail (Fig. 2B).

### WB analysis of EM-G3 cells

**Figure 3.**
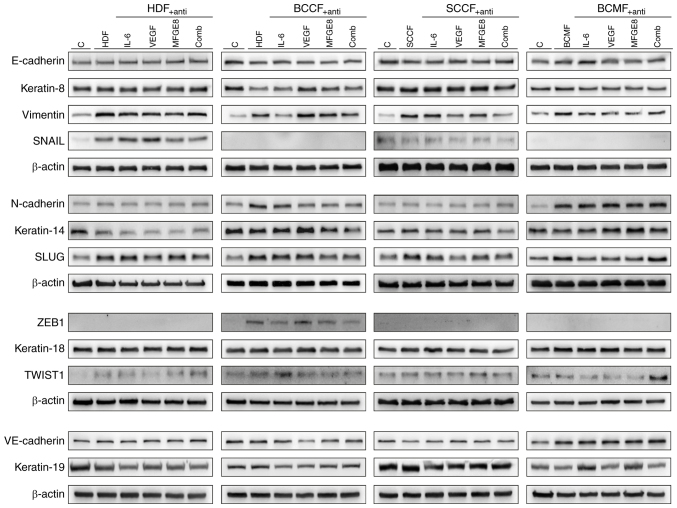
Western blot analysis of EM-G3 cells. Protein expression analysis in indirect (CM) co-culture of EM-G3 cells with HDFs, BCCFs, SCCFs and BCMFs. The studied conditions for each studied fibroblast type included: Control culture of EM-G3 cells, EM-G3 cells cultured in CM derived from respective fibroblasts, EM-G3 cells cultured in CM enriched with neutralizing antibodies against either IL-6 or VEGF-A or MFGE8 or combination of all three tested antibodies (anti-IL-6 + anti-VEGF-A + anti-MFGE8). The expression profile of the following markers related to cell differentiation and epithelial-to-mesenchymal transition was evaluated: keratin-8, keratin-14, keratin-18, keratin-19, vimentin, SLUG, SNAIL, TWIST1, ZEB1, E-cadherin, N-cadherin, VE-cadherin. β-Actin was used as sample loading control (representative beta-actin controls are shown). HDFs, human dermal fibroblasts; BCCFs, basal cell carcinoma fibroblasts; SCCFs, squamous cell carcinoma fibroblasts; BCMFs, breast cancer metastasis fibroblasts; CM, conditioned media.

WB analysis of EM-G3 was performed, cells cultured under control conditions and following indirect co-culture with CM from HDFs, BCCFs, SCCFs and BCMFs (Fig. 3). The expression levels of keratins-8, −14, −18 and −19 were investigated, reflecting cell differentiation as well as key markers associated with epithelial-mesenchymal transition (EMT) such as TWIST1, SLUG, SNAIL and ZEB1. Additionally, the expression of E-cadherin and N-cadherin were assessed to explore epithelial-mesenchymal phenotypic transitions, and vimentin as a mesenchymal marker. VE-cadherin expression was also examined as an indicator of invasiveness. The effects of neutralizing antibodies were investigated, which target IL-6, VEGF-A and MFGE8, both individually and in combination, to attenuate the effects of CM.

The analysis in the present study revealed moderate expression of keratins (keratin-8, −14, −18, and −19) in EM-G3 cells under control conditions. Positive expression of SLUG, SNAIL and TWIST1 among the EMT-associated transcription factors were observed. Vimentin, a mesenchymal marker, was weakly expressed in EM-G3 cells. Notably, EM-G3 cells exhibited positive expression of cadherins (E-cadherin, N-cadherin and VE-cadherin), with higher expression of E-cadherin compared with N-cadherin.

The co-culture experiments demonstrated heterogeneous effects of the tested fibroblasts on the keratin expression pattern in EM-G3 cells. Treatment with CM-HDF downregulated keratins-14 and −19, while CM-BCCF downregulated keratins-8 and −14. Interestingly, supplementation with CM-HDF led to increased keratin-8 expression and decreased keratin-19 expression. Moreover, supplementation with CM-BCCF attenuated the negative effect on keratin-8 expression and further decreased keratin-14 expression. By contrast, CM-SCCF and CM-BCMF upregulated keratin-14 and keratins-8/-18, respectively. The stimulatory effects of CM-SCCF on keratin-14 and CM-BCMF on keratins-8/-18 in EM-G3 cells were partially attenuated following supplementation with neutralizing antibodies.

Co-culturing EM-G3 cells with CM derived from all studied fibroblasts led to upregulation of vimentin, a mesenchymal marker. Supplementation of CM with neutralizing antibodies attenuated this effect, particularly in CM-SCCF. Expression of SLUG was stimulated by all studied CM. The combination of neutralizing antibodies was able to diminish this effect, except for CM-BCMF.

The weak expression of TWIST1 remained stable in all tested conditions. Interestingly, CM derived from HDFs and SCCFs activated SNAIL expression, whereas CM-BCCF was the only treatment able to activate ZEB1 expression. The tested combination of neutralizing antibodies was able to attenuate only the increased expression of ZEB1.

Expression of E- and N-cadherins was not affected by CM derived from HDFs. CM-BCCF clearly induced an E- to N-cadherin switch, which was partially attenuated by supplementation with neutralizing antibodies. By contrast, CM-SCCFs only weakly decreased expression of E-cadherin, and this effect was diminished by supplementation. CM-BCMF increased N-cadherin expression, with no response to supplementation with neutralizing antibodies. The expression of VE-cadherin was only increased in response to CM derived from BCMFs and was not affected by neutralizing antibodies.

### IF analysis of EM-G3 cells

**Figure 4.**
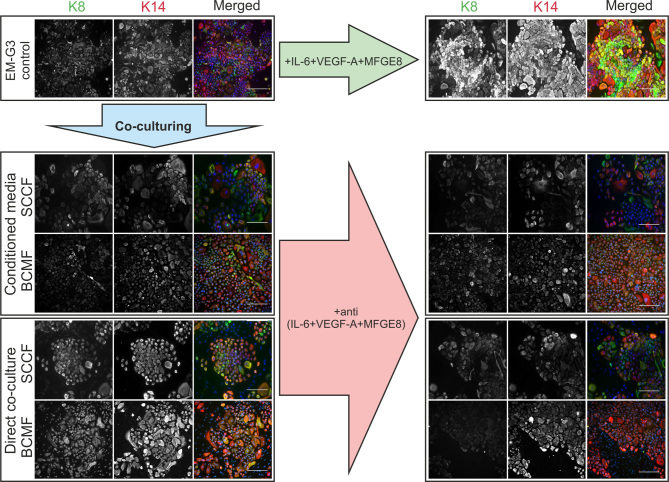
Immunofluorescence analysis EM-G3 cells. Expression of keratin-8 and keratin-14 in EM-G3 cells directly and indirectly (conditioned media) co-cultured with HDFs, SCCFs, and BCMFs. The co-expression of keratins-8/-14 was increased in EM-G3 cells co-cultured with cancer-associated fibroblasts. Addition of neutralizing antibodies against IL-6, VEGF-A, and MFGE8 attenuated the co-expression of keratins-8/-14. Positive control included supplementation of culture media with human recombinant IL-6, VEGF-A, and MFGE8 and resulted in marked co-expression of keratins-8/-14. HDFs, human dermal fibroblasts; SCCFs, squamous cell carcinoma fibroblasts; BCMFs, breast cancer metastasis fibroblasts.

IF analysis confirmed that EM-G3 cells were positive for both examined markers (Fig. 4), namely keratin-8 and keratin-14, thus correlating with WB. Furthermore, the microscopic examination revealed weak co-expression (that is not possible to evaluate by WB) of keratins-8 and −14 in control culture of EM-G3 cells. Both direct and indirect co-culturing of EM-G3 cells with fibroblasts resulted in increased numbers of keratins-8/-14 co-expressing cells. Importantly, this effect appeared more pronounced in CAF co-cultures when compared with HDFs. Upon the addition of neutralizing antibodies, a notable decrease was observed in keratin-8/-14 co-expression.

To complete the panel of experiments, recombinant proteins (IL-6 + VEGF-A + MFGE8) were also added to cultures of EM-G3 cells as positive control, leading to an excessive increase of keratin-8/-14 co-expressing populations.

## Discussion

The present study demonstrated the effect of CAFs on EM-G3 cells, which serve as representative of breast cancer cells. Regardless of their origin, all types of CAFs expressed high levels of *IL6, VEGFA* and *MFGE8* genes. The products of these genes contribute to the establishment of an inflammatory microenvironment and promote vascularization, which are known to be positively associated with cancer initiation and progression (37,38). Targeting CAFs has emerged as an increasingly appealing approach for cancer therapy (14,39). The WB analysis showed positivity for keratins-8, −14, −18 and −19 on EM-G3 cells, although keratin-19 expression was not originally reported during establishment of this cell line (23). The IF staining revealed two major distinct populations expressing either keratin-8 or keratin-14, along with a minor population co-expressing keratin-8/-14. Such observation is in accordance with the previously reported ‘progenitor’ character of EM-G3 cells. In detail, mammary stem cells (keratin-14^+^, keratin-19^+^) give rise to transit amplifying cells expressing both luminal (keratin-8^+^, keratin-19^+^) and basal/myoepithelial (keratin-14^+^) markers (40). Direct and in-direct co-culture of EM-G3 cells with fibroblasts resulted in outgrowth of a keratin-8/-14 co-expressing population particularly in CAF co-cultures. This effect may result from the specific interaction between epithelial and stromal cell populations. CAFs represent the most abundant and bio-active non-cancer stromal cells of the breast TME (41). Furthermore, CAFs dramatically sustain breast cancer progression by interacting with cancer cells. Additionally, the detection of breast-CAF aggregates in the peripheral blood of patients with metastatic disease leads one to speculate that these aggregates could represent an additional marker of breast cancer metastasis and could influence the metastatic process (42).

Although CAFs are described as constitutively activated cells, several studies have demonstrated that it is possible to normalize CAFs or inhibit their biological functions through the administration of specific CAF-targeting agents (21). Since the authors previously found a similar stimulatory effect on the generation of a keratin-8/-14 co-expressing population by CAFs isolated from different solid tumors (22), it was further hypothesized that the transcriptomic comparison of the CAF gene expression profile could select overlapping candidate genes that may have the potential to target the breast cancer microenvironment. In this context, IL-6, VEGF-A and MFGE8 were selected (in combination, rather than individually) and found to be effective in eliminating the keratin-8/-14 population in the used co-culture *in vitro* systems. In parallel, WB analysis was also conducted of keratin expression to reveal whether the expression pattern changes induced by CM derived from different fibroblasts that are affected by the presence of tested neutralizing antibodies. The keratin-8/-18 presence on breast cancer cell lines correlated with a less invasive phenotype and the absence correlated with a highly invasive, dedifferentiated phenotype (43). On the other hand, collective invasion in breast cancer can be initiated by cancer cells that express basal epithelial markers, with keratin-14 being a notable representative of these markers (44,45). CM derived from BCCFs was able to decrease keratin-8 expression; an effect partially reverted by the combination of neutralizing antibodies used. Intriguingly, CM-BCMF rather increased the total amount of keratin-8, which may be related to the metastatic origin of these fibroblasts. However, acquiring a sample from the primary tumor of breast cancer for fibroblast isolation remains difficult due to the necessity of conducting a comprehensive pathological examination of the entire tumor to accurately determine staging (46). This inability represents a certain limitation to the present study.

Moreover, vimentin plays an important role in the promotion of breast cancer cell migration and invasion (47). In this context, it was observed that the rather weak expression of vimentin, a mesenchymal marker associated with poor prognosis in breast cancer (48,49), was markedly stimulated by co-culturing EM-G3 cells with fibroblasts. Importantly, a combination of neutralizing antibodies attenuated this effect to a various extent depending on the fibroblast origin. The expression of commonly studied EMT markers (SLUG, SNAIL, TWIST1, ZEB1) correlated well with the poor prognosis in breast cancer patients (50,51). In this context, it was observed as a clinically relevant normalizing effect of the tested neutralizing antibody combination on SLUG and ZEB1 expression in EM-G3 cells induced by fibroblasts.

Finally, co-culturing (and targeting with neutralizing antibodies) of EM-G3 cells modulates the expression of cadherins was assessed. The expression of both E- and N-cadherin was maintained in both the primary tumors and metastatic lesions. Both BCCF and SCCF co-culturing with EM-G3 cells decreased expression of E-cadherin, whereas BCMF co-culturing led to a slight increase in its expression. Although it has been shown that the breast cancer-specific mortality was unrelated to E-cadherin expression in multiple models, E-cadherin low expression has been associated with lobular histology, tumor characteristics and menopausal hormone use, with no evidence of an association with breast cancer-specific survival. These findings underscore the significance of reduced E-cadherin expression as a marker for specific tumor subtypes (52). However, both BCCFs and BCMFs strongly increased N-cadherin expression. It has been demonstrated that N-cadherin promotes motility, invasion and metastasis even in the presence of the normally suppressive E-cadherin (53). From this point of view, the overall outcome of the EM-G3 interactions with CAFs resulted in a cancer-promoting phenotype. In detail, N-cadherin-expressing cells increase MMP-9 production responsible for greater ability to penetrate matrix protein barriers, while the increase in their adherence to endothelium may improve their ability to enter and exit the vasculature (53). The changes in E-/N-cadherin expression in EM-G3 cells induced by fibroblasts were positively improved by the tested combination of neutralizing antibodies, suggesting t another clinically relevant implication. Apart from N-cadherin, BCMFs also increased expression of VE-cadherin, which was shown to promote invasiveness by increasing the adhesion of breast cancer cells to the endothelium and is involved in the initial phase of incorporation, but not in their transmigration (54). Notably, all conclusions were solely drawn from EM-G3 cells and constrained availability of biological replicates of CAFs, what represents a certain limitation to the present study. It would be valuable to replicate critical findings also using other (pre)malignant breast cancer cell lines and CAFs isolated from further patients to enhance the robustness and statistical reliability of the results. Additionally, it is worth noting that proliferation tests were not conducted, which could provide further insights into cellular behaviours. The authors acknowledge the potential of the present study's results in advancing the understanding of the TME, particularly regarding influence of CAFs on poor prognosis markers. The present study lays the groundwork for targeted therapies by identifying IL-6, VEGF-A and MFGE8 as potential targets based on transcriptomic profiling. While the present study focuses on EM-G3 cells, extrapolating to other breast cancer cell lines and stages of tumorigenesis requires further exploration.

In conclusion, the present study sheds light on the intricate interactions between CAFs and breast cancer EM-G3 cell line. While recognizing the need for further investigations, several clinically relevant outcomes have emerged. A tumor-promoting effect of CAFs on EM-G3 cells was observed, highlighting the potential heterogeneity within CAF populations. This heterogeneity, as described in breast cancer (55), underscores the importance of selecting optimal treatment strategies, especially for metastatic disease with a poor prognosis (56). Moreover, the findings of the present study suggested that regardless of their origin, CAFs express genes that promote inflammation, a critical factor in cancer progression. These insights pave the way for future studies delving into the specific interactions between distinct CAF (sub)populations and breast cancer cells, with implications for metastasis facilitation and the development of clinically relevant treatment protocols. Notably, the use of a combined neutralizing antibodies against specific proteins, guided by transcriptomic profiling of CAFs, effectively attenuated the modulatory effect of CAFs on epithelial breast cancer cells. This supports the rationale for therapeutic targeting of CAFs as a promising avenue in the management of various cancer types. While the present study serves as a stepping stone in understanding these complexities, it emphasizes the need for ongoing research to fully comprehend the multifaceted relationship between CAFs and breast cancer, ultimately benefiting patient care.

## Supplementary Material

Supporting Data

Supporting Data

## Figures and Tables

**Table I.** Enzyme-linked immunosorbent assay (ELISA) kits used for the evaluation of IL-6, VEGF-A and MFGE8 production by fibroblasts into culture media.

Recombinant protein		Cat. no.	Produced by
h-recombinant VEGF-A		RBMS277/2R	BioVendor R&D
h-recombinant IL-6		RBMS231/2R	BioVendor R&D
h-recombinant MFGE-8		E91286Hu	Wuhan USCN Business Co., Ltd.

**CAFs**	**IL-6**	**VEGF-A**	**MFGE8**

SCCFs	11.0±4.0 pg	49.3±13.5 pg	106.1±24.0 pg
BCCFs	6.3±0.7 pg	51.8±10.1 pg	90.2±1.5 pg
BCMFs	7.4±1.6 pg	31.1±12.4 pg	115.2±36.9 pg

CAFs, cancer associated fibroblasts; SCCFs, squamous cell carcinoma fibroblasts; BCCFs, basal cell carcinoma fibroblasts; BCMFs, breast cancer metastasis fibroblasts.

**Table II.** Humanized recombinant proteins and blocking antibodies used in the cell culture experiment.

Recombinant protein/blocking antibody	Cat. no	Produced by
h-recombinant VEGF-A	293-VE-050	R&D Systems, Inc.
h-recombinant IL-6	206-IL	R&D Systems, Inc.
h-recombinant MFGE8	2767-MF-050	R&D Systems, Inc.
anti-VEGF-A	MAB-293	R&D Systems, Inc.
anti-IL-6	AF-206-NA	R&D Systems, Inc.
anti-MFGE8	11-113-C100	EXBIO Praha, a.s.

**Table III.** Reagents used for immunofluorescent staining of cells.

Primary antibody	Host	Isotype	Dilution	Cat. no.	Clonality	Produced by
α-smooth muscle actin	Mouse	IgG	1:100	M0851	Monoclonal	Dako; Agilent Technologies, Inc.
Tenascin-C	Rabbit	IgG	1:200	AB19013	Monoclonal	Sigma-Adrich; Merck KGaA
Fibronectin	Rabbit	IgG	1:100	A0245	Polyclonal	Dako; Agilent Technologies, Inc.
Keratin-8	Mouse	IgG	1:100	MA5-14428	Polyclonal	Sigma-Adrich; Merck KGaA
Keratin-14	Mouse	IgG	1:100	PA5-28002	Monoclonal	Sigma-Adrich; Merck KGaA
Ki67	Mouse	IgG	1:100	MIB-1	Monoclonal	Dako; Agilent Technologies, Inc.
Wide Spectrum Cytokeratin	Rabbit	IgG	1:50	SAB3705	Polyclonal	Abcam
Vimentin	Mouse	IgG	1:100	M0725	Polyclonal	Dako; Agilent Technologies, Inc.

**Secondary antibody**	**Host**	**Isotype**			**Clonality**	**Produced by**

Goat anti-mouse IgG	Goat	IgG	1:300	T5393	Polyclonal	Sigma-Adrich; Merck KGaA
(TRITC-conjugated)						
Swine anti-rabbit	Swine		1:300	F0054	Polyclonal	Dako; Agilent Technologies, Inc.
Immunoglobulins (FITC conjugated)						
Anti mouse IgG, Alexa	Goat	IgG	1:300	A32723	Polyclonal	Invitrogen; Thermo Fisher
Fluor™ Plus 488						Scientific, Inc.
Anti-rabbit IgG, Alexa	Goat	IgG	1:300	A32740	Polyclonal	Invitrogen; Thermo Fisher
Fluor™ Plus 594						Scientific, Inc.

**Table IV.** Reagents used for western blot staining of cells.

Primary antibody	Host	Isotype	Clonality	Dilution	Cat. no.	Produced by
E-cadherin	Rabbit	IgG	Monoclonal	1:1,000	3195	Cell Signaling Technology, Inc.
N-cadherin	Rabbit	IgG	Monoclonal	1:1,000	13116	Cell Signaling Technology, Inc.
VE-cadherin	Rabbit	IgG	Polyclonal	1:1,000	ab33168	Abcam
Keratin-8	Rabbit	IgG	Monoclonal	1:10,000	ab53280	Abcam
Keratin-14	Rabbit	IgG	Monoclonal	1:20,000	ab51054	Abcam
Keratin-18	Rabbit	IgG	Monoclonal	1:10,000	ab133302	Abcam
Keratin-19	Mouse	IgG	Monoclonal	1:200	MA5-12663	Invitrogen; Thermo Fisher Scientific, Inc.
SLUG	Rabbit	IgG	Monoclonal	1:1,000	9585	Cell Signaling Technology, Inc.
SNAIL	Mouse	IgG	Monoclonal	1:500	14-9859-82	eBioscience; Thermo Fisher Scientific, Inc.
TWIST1	Rabbit	IgG	Monoclonal	1:1,000	69366	Cell Signaling Technology, Inc.
ZEB1	Rabbit	IgG	Monoclonal	1:1,000	70512	Cell Signaling Technology, Inc.
Vimentin	Rabbit	IgG	Monoclonal	1:1,000	5741	Cell Signaling Technology, Inc.
β-actin	Rabbit	IgG	Monoclonal	1:1,000	8457	Cell Signaling Technology, Inc.

**Secondary antibody**	**Host**	**Isotype**		**Dilution**	**Cat. no.**	**Produced by**

Anti-rabbit IgG, HRP-linked	Goat	IgG		1:1,000	7074	Cell Signaling Technology, Inc.
Anti-mouse	Horse	IgG		1:1,000	7076	Cell Signaling Technology, Inc.
IgG, HRP-linked						

## Data Availability

The microarray datasets generated and/or analyzed during the current study are available in MIAME (Minimum Information About a Microarray Experiment) compliant format in the ArrayExpress database (accession no. E-MTAB-12994). The datasets used and/or analyzed during the current study are available from the corresponding author on reasonable request.
