# Supplementary material for: Unravelling heterogeneous effects of cancer‑associated fibroblasts on poor prognosis markers in breast cancer EM‑G3 cell line: In vitro‑targeted treatment (anti‑IL-6, anti‑VEGF-A, anti‑MFGE8) based on transcriptomic profiling
Source: Oncol Rep. 2023 Nov 15;51(1):3. doi: 10.3892/or.2023.8662 (PMC10688412; doi:10.3892/or.2023.8662)

Figure S1. Multicolor fluorescence *in situ* hybridization (mFISH) analysis of EM-G3 cells. The analysis confirmed a stable diploid genome with several genetic changes. Two karyotypes were identified: (A) 44,XX,der(3)t(3;9)(q26;?),der(6)t(6;15)(p21;q12),der(12)t(12;12)(p13.2;q?)dup(12)(q21.2q21.3)dup(12)(q21.2q21.3),der(13)t(13;20)(p11;?),-15,-20. (B) 45,idem,+der(7)t(7;12)(q11;?).

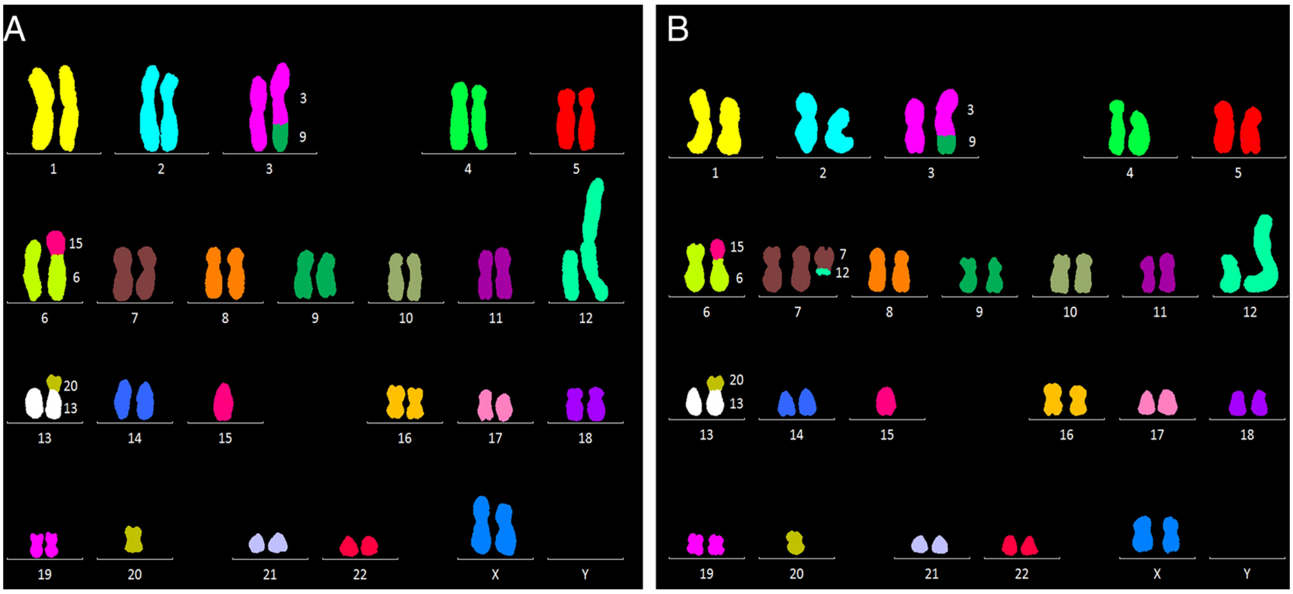

Supplement: Supporting Data [file Supplementary_Data1.pdf]
