# Supplementary material for: Unravelling heterogeneous effects of cancer‑associated fibroblasts on poor prognosis markers in breast cancer EM‑G3 cell line: In vitro‑targeted treatment (anti‑IL-6, anti‑VEGF-A, anti‑MFGE8) based on transcriptomic profiling
Source: Oncol Rep. 2023 Nov 15;51(1):3. doi: 10.3892/or.2023.8662 (PMC10688412; doi:10.3892/or.2023.8662)
Supplement: Supporting Data [file Supplementary_Data2.pdf]

Table SI. List of common upregulated genes defined as the genes with logFC > 0.8 in all comparisons and with F-test FDR value less than 0.05.

**Gene symbol**	**Gene name**	**Illumina Probe ID**	**Average expression log2-intensity**	**Log2 fold change SCCF - HF_der**	**Log2 fold change MEBF - HF_der**	**Log2 fold change BCCF - HF_der**	**F-test P-value**	**F-test FDR**
GPR177	Wnt ligand secretion mediator	ILMN_1671260	9,62	4,78	4,84	0,81	$1,90x10^{-16}$	$3x10^{-13}$
TMTC1	transmembrane O-mannosyltransferase targeting cadherins 1	ILMN_1811426	7,54	4,94	5,48	1,05	$7,2x10^{-16}$	$9,5x10^{-13}$
IGFBP7	insulin like growth factor binding protein 7	ILMN_2062468	12,34	3,55	3,79	2,79	$2,4x10^{-15}$	$2,5x10^{-12}$
OSBPL10	oxysterol binding protein like 10	ILMN_1669497	6,56	2,42	3,58	0,93	$3,2x10^{-13}$	$1,8x10^{-10}$
CDH6	cadherin 6	ILMN_1766675	8,69	5,72	5,46	1,75	$4,30x10^{-11}$	$1,5x10^{-8}$
TBX2	T-box transcription factor 2	ILMN_1792256	6,67	3,23	2,79	1,12	$4,2x10^{-10}$	$1,1x10^{-7}$
EFEMP1	EGF containing fibulin extracellular matrix protein 1	ILMN_2350634	10,62	4,85	5,54	3,97	$1,6x10^{-9}$	$3,4x10^{-7}$
C19orf4	transmembrane protein 59 like	ILMN_1730945	7,03	2,63	5,04	1,5	$2,8x10^{-}7$	$2,6x10^{-5}$
KCNK6	potassium two pore domain channel subfamily K member 6	ILMN_1701173	7,8	1,02	1,22	1,88	$3,7x10^{-7}$	$3,4x10^{-5}$
RDH10	retinol dehydrogenase 10	ILMN_1714335	9,63	3,45	3,36	1,85	$4x10^{-7}$	$3,5x10^{-5}$
ARHGAP20	Rho GTPase activating protein 20	ILMN_1782761	5,77	1,83	2,23	0,93	$8,2x10^{-7}$	$5,9x10^{-5}$
PTGFRN	prostaglandin F2 receptor inhibitor	ILMN_1743130	8,35	1,89	1,9	0,96	$8,3x10^{-7}$	$6x10^{-5}$
CRLF1	cytokine receptor like factor 1	ILMN_1681515	8,56	2,32	5,15	2,4	$8,5x10^{-7}$	$6x10^{-5}$
MFGE8	milk fat globule EGF and factor V/VIII domain containing	ILMN_1756071	13,92	1,47	2,31	1,66	$8,7x10^{-7}$	$6,2x10^{-5}$
TPD52L1	TPD52 like 1	ILMN_1714383	6,63	1,54	1,27	2,53	$1,3x10^{-6}$	$8,4x10^{-5}$
GRAMD3	GRAM domain containing 2B	ILMN_2065690	10,23	1,35	2,19	1,92	$1,4x10^{-6}$	$9x10^{-5}$

LEPREL1	prolyl 3-hydroxylase 2	ILMN_1657373	9,55	3,51	4,01	0,89	$1,4x10^{-6}$	$9,20x10^{-5}$
RAMP1	receptor activity modifying protein 1	ILMN_1764754	6,56	2,46	3,72	1,67	$2x10^{-6}$	0,00012
FLJ10916	threonine synthase like 2	ILMN_2173294	8,2	2,3	2,63	2,59	$2,2x10^{-6}$	0,00013
RARRES3	phospholipase A and acyltransferase 4	ILMN_1701613	6,9	2,09	1,55	1,14	$2,6x10^{-6}$	0,00015
PRSS23	serine protease 23	ILMN_1797776	9,08	1,65	1,45	1,91	$3,6x10^{-6}$	$2x10^{-4}$
FOXC2	forkhead box C2	ILMN_1705201	6,05	1,96	1,5	0,9	$4,1x10^{-6}$	0,00022
IL6	interleukin 6	ILMN_1699651	8,18	3	1,35	3,88	$5,5x10^{-6}$	0,00028
IGFBP4	insulin like growth factor binding protein 4	ILMN_1665865	12,94	1,87	1,44	0,86	$7x10^{-6}$	0,00034
RPS6KA2	ribosomal protein S6 kinase A2	ILMN_1702501	10,41	0,97	1,34	1,61	$7,1x10^{-6}$	0,00034
TFG	trafficking from ER to golgi regulator	ILMN_2341815	10,5	1,87	2,3	2,15	$7,2x10^{-6}$	0,00034
LAMC1	laminin subunit gamma 1	ILMN_1810852	12,45	1,19	1,17	1,15	$7,5x10^{-6}$	0,00035
VLDLR	very low density lipoprotein receptor	ILMN_2361862	6,51	1,23	2,81	0,82	$7,8x10^{-6}$	0,00036
JAG1	jagged canonical Notch ligand 1	ILMN_1691376	6,61	1,96	3,38	2,01	$8,3x10^{-6}$	0,00039
RGS4	regulator of G protein signaling 4	ILMN_1758067	9,27	2,37	1,86	1,51	$9x10^{-6}$	0,00041
CHIC2	cysteine rich hydrophobic domain 2	ILMN_1679428	10,61	1,04	1,06	0,82	1x10-5	0,00045
MGST1	microsomal glutathione S-transferase 1	ILMN_2355168	11,43	1,87	1,92	1,65	$1,4x10^{-5}$	0,00057
ITGAV	integrin subunit alpha V	ILMN_2169439	9,8	1,12	1,68	0,93	$1,7x10^{-5}$	0,00067
SFRP4	secreted frizzled related protein 4	ILMN_1810172	6,7	1,39	3,47	3,95	$1,8x10^{-5}$	0,00073
NTN4	netrin 4	ILMN_2113490	7	2,6	3,6	0,84	$1,9x10^{-5}$	0,00075
SUSD2	sushi domain containing 2	ILMN_1693270	7,86	3,86	3,62	2,23	$2,2x10^{-5}$	0,00085
LOC284023	ring finger protein 227	ILMN_1739325	7,4	1,45	1,99	1,13	$2,4x10^{-5}$	0,00089
CXCL16	C-X-C motif chemokine ligand 16	ILMN_1728478	6,68	1,74	2,43	1,17	$2,6x10^{-5}$	0,00098
CCPG1	NA	ILMN_1794190	8,61	0,92	1,43	1,42	$3,1x10^{-5}$	0,0011
MSC	musculin	ILMN_1741404	7,75	1,94	2,64	1,69	$3,1x10^{-5}$	0,0011
NOV	cellular communication network factor 3	ILMN_1787186	7,46	1,83	2,85	1,07	$3,7x10^{-5}$	0,0013

PDGFRL	platelet derived growth factor receptor like	ILMN_1680339	7,08	2,47	4,27	0,98	$4,2x10^{-5}$	0,0014
IGFBP2	insulin like growth factor binding protein 2	ILMN_1725193	8,01	2,46	2,75	3,58	$4,3x10^{-5}$	0,0014
F2R	coagulation factor II thrombin receptor	ILMN_1742866	9,16	1,51	1,02	0,9	$5x10^{-5}$	0,0016
PNMA2	PNMA family member 2	ILMN_1790778	6,25	2,48	1,59	1,44	$5,1x10^{-5}$	0,0017
DEPDC6	DEP domain containing MTOR interacting protein	ILMN_1756685	6,61	2,64	3,5	2,1	$5,3x10^{-5}$	0,0017
MFAP5	microfibril associated protein 5	ILMN_1733415	10,44	1,67	0,99	3,64	$5,9x10^{-5}$	0,0018
SLC1A1	solute carrier family 1 member 1	ILMN_1658917	6,78	2,03	1,75	0,85	$6,5x10^{-5}$	0,002
HAPLN3	hyaluronan and proteoglycan link protein 3	ILMN_1654319	6,71	0,9	1,94	2,48	$7,8x10^{-5}$	0,0023
DIO2	iodothyronine deiodinase 2	ILMN_1737650	6,78	3,23	4,18	1,7	$8,5x10^{-5}$	0,0025
FZD4	frizzled class receptor 4	ILMN_1743367	8,51	2,4	1,67	0,96	$1x10^{-4}$	0,0029
VEGFA	vascular endothelial growth factor A	ILMN_2375879	7,51	0,89	1,06	2,05	0,00012	0,0032
COL4A1	collagen type IV alpha 1 chain	ILMN_1653028	10,19	0,84	1,3	1,92	0,00015	0,004

Table SII. List of common downregulated genes defined as the genes with logFC < -0.8 in all comparisons and with F-test FDR value less than 0.05.

**Gene symbol**	**Gene name**	**Illumina Probe ID**	**Average expression log2-intensity**	**Log2 fold change SCCF - HF_der**	**Log2 fold change MEBF - HF_der**	**Log2 fold change BCCF - HF_der**	**F-test P-value**
PODXL	podocalyxin like	ILMN_2413158	8,7	-5,66	-7,41	-1,09	1,20x10^{-21}
HOXB8	homeobox B8	ILMN_1798496	6,32	-5,04	-5,1	-1,85	1,3x10^{-20}
C4orf31	neuron derived neurotrophic factor	ILMN_1689176	6,52	-4,5	-4,95	-2,04	1,2x10^{-12}
AKR1C2	aldo-keto reductase family 1 member C2	ILMN_2412336	8,58	-2,66	-1,33	-0,89	1,5x10^{-11}
CD24	CD24 molecule	ILMN_2060413	6,08	-4,7	-5,02	-3,11	2x10^{-}11
QPRT	quinolinate phosphoribosyltransferase	ILMN_1700268	6,81	-3,27	-3,53	-2,17	6,9x10^{-11}
BAALC	BAALC binder of MAP3K1 and KLF4	ILMN_1687751	7,85	-3,45	-2,38	-1,17	6x10^{-10}
GNG2	G protein subunit gamma 2	ILMN_1807925	5,91	-3,14	-3,12	-1,66	7,4x10^{-10}
MATN2	matrilin 2	ILMN_1694840	8,58	-2,94	-3,02	-1,71	9,3x10^{-10}
MMP12	matrix metallopeptidase 12	ILMN_2073758	5,06	-2,22	-2,34	-1,74	2x10^{-9}
PIK3R1	phosphoinositide-3-kinase regulatory subunit 1	ILMN_1760303	8,05	-1,39	-2,02	-0,8	2,9x10^{-9}
CGB5	chorionic gonadotropin beta 5 subunit	ILMN_2163790	5,05	-2,15	-2,11	-1,79	6,2x10^{-9}
PTPRN	protein tyrosine phosphatase receptor type N	ILMN_1658576	5,31	-2,59	-2,77	-1,88	3,4x10^{-8}
MMP3	matrix metallopeptidase 3	ILMN_1784459	8,39	-4,45	-6,43	-1,96	4,8x10^{-8}

C11orf87	chromosome 11 open reading frame 87	ILMN_1810376	5,29	-2,23	-2,43	-1,17	$5{,}9x10^{-8}$
ANPEP	alanyl aminopeptidase, membrane	ILMN_1763837	10,9	-1,61	-1,58	-0,88	$1{,}2x10^{-7}$
MOXD1	monooxygenase DBH like 1	ILMN_1687501	9,71	-2,2	-1,75	-1,15	$1{,}7x10^{-}7$
TRNP1	TMF1 regulated nuclear protein 1	ILMN_1695946	11,36	-2,02	-2,13	-1,48	$4{,}4x10^{-7}$
LRP4	LDL receptor related protein 4	ILMN_1675268	6,26	-2,62	-1,55	-0,88	$7{,}1x10^{-7}$
CGB1	chorionic gonadotropin subunit beta 1	ILMN_2044645	4,9	-1,44	-1,5	-1,41	$8{,}4x10^{-7}$
PRLR	prolactin receptor	ILMN_1669781	5	-1,67	-1,8	-1,42	$8{,}9x10^{-7}$
KCNS3	potassium voltage-gated channel modifier subfamily S member 3	ILMN_2175112	7,04	-1,3	-2,02	-1,81	$9{,}9x10^{-7}$
HAND1	heart and neural crest derivatives expressed 1	ILMN_1680987	5,27	-2,12	-2,52	-1,78	$1{,}1x10^{-6}$
ZNF536	zinc finger protein 536	ILMN_1772155	6,29	-2,48	-1,61	-2	$2{,}1x10^{-6}$
EDG1	sphingosine-1-phosphate receptor 1	ILMN_1653504	5,82	-1,8	-1,41	-1,12	$2{,}1x10^{-6}$
TSPAN13	tetraspanin 13	ILMN_1669881	7,79	-2,58	-3,69	-1,86	$2{,}8x10^{-6}$
CPEB1	cytoplasmic polyadenylation element binding protein 1	ILMN_2369603	5,25	-1,58	-1,19	-1,5	$3{,}1x10^{-6}$
HOMER2	homer scaffold protein 2	ILMN_1671486	6,29	-2,47	-1,24	-1,2	$3{,}5x10^{-6}$
GRP	gastrin releasing peptide	ILMN_2413323	4,85	-2,1	-2,14	-1,99	$5{,}5x10^{-6}$
TSPAN10	tetraspanin 10	ILMN_1656194	7,11	-1,03	-1,25	-1,79	$7{,}5x10^{-6}$

CNIH3	cornichon family AMPA receptor auxiliary protein 3	ILMN_1749071	6,11	-1,72	-2,7	-0,86	$8,6x10^{-6}$
SOX11	SRY-box transcription factor 11	ILMN_1773459	4,7	-0,99	-1,14	-0,9	$1,3x10^{-5}$
TRPV2	transient receptor potential cation channel subfamily V member 2	ILMN_2049536	8,06	-3,38	-1,85	-1,87	$1,6x10^{-5}$
CCDC85A	coiled-coil domain containing 85A	ILMN_1669982	6,96	-2,73	-3,96	-1,28	$1,6x10^{-5}$
NETO2	neuropilin and tolloid like 2	ILMN_1760849	6,69	-2,2	-3,43	-1,63	$2,1x10^{-5}$
TRIM55	tripartite motif containing 55	ILMN_2347622	4,94	-1,29	-1,73	-1,57	$2,2x10^{-5}$
SSX2IP	SSX family member 2 interacting protein	ILMN_1720844	6,44	-2,07	-2,72	-1,62	$2,2x10^{-5}$
CGNL1	cingulin like 1	ILMN_1730229	5,69	-1,74	-1,7	-1,18	$2,4x10^{-5}$
HOXA5	homeobox A5	ILMN_1753613	5,07	-2,04	-2,06	-1,25	$2,9x10^{-5}$
ST8SIA2	ST8 alpha-N-acetyl-neuraminide alpha-2,8-sialyltransferase 2	ILMN_1758461	5,29	-1,96	-2,17	-0,94	$3,1x10^{-5}$
HEY1	hes related family bHLH transcription factor with YRPW motif 1	ILMN_1788203	5,54	-1,76	-2,15	-1,89	$3,3x10^{-5}$
DTNA	dystrobrevin alpha	ILMN_1730201	6,29	-1,35	-1,93	-1,39	$3,4x10^{-5}$
CXCL12	C-X-C motif chemokine ligand 12	ILMN_1791447	10,95	-3,42	-2,66	-1,13	$3,6x10^{-5}$
TYRO3	TYRO3 protein tyrosine kinase	ILMN_1740169	7,69	-0,86	-1,61	-0,83	$3,7x10^{-5}$

CLEC12A	C-type lectin domain family 12 member A	ILMN_2292178	4,74	-1,38	-1,37	-1,14	$4{,}9x10^{-5}$
IFI44	interferon induced protein 44	ILMN_1760062	6,75	-2,02	-1,41	-0,87	$6{,}1x10^{-5}$
ZNF536	zinc finger protein 536	ILMN_2150586	4,93	-1,28	-0,92	-1,05	$6{,}2x10^{-5}$
AMPH	amphiphysin	ILMN_1685834	8,4	-1,54	-1,33	-1,06	$8{,}2x10^{-5}$
NKX2-6	NK2 homeobox 6	ILMN_1766678	4,8	-1,05	-1,16	-1,01	0,00011

Table SIII. Results of the functional enrichment analysis performed by Enricher web server on upregulated genes (shown in Table SI).

Term	P-value	Adjusted P-value	Odds Ratio	Combined Score	Assigned Genes
NOTCH Signaling in Multiple Myeloma	<0.0001	0.001	108.7	1302.8	*IL6*<br>JAG1<br>*VEGFA*
VEGFA/NOTCH1/WNT Cross-talk in Blood Vessel Sprouting and Branching in Cancer	<0.0001	0.001	47.8	607.5	FOXC2<br>JAG1<br>FZD4<br>*VEGFA*
Coagulation Factors and PAR1/2 Receptors (F2R/F2RL1) in Endometriosis	< 0.0001	0.009	41.2	387.5	*IL6*<br>F2R<br>*VEGFA*
Tumor Infiltrating Macrophages in Cancer Progression and Immune Escape	0.0001	0.009	36.2	327.5	*IL6*<br>*MFGE8*<br>VEGFA
Mast-Cell Activation without Degranulation	0.0003	0.015	26.0	210.8	*IL6*<br>F2R

					VEGFA
